# Rolling membrane powered by low-temperature steam as a new approach to generate mechanical energy

**DOI:** 10.1038/s41598-020-73732-7

**Published:** 2020-10-06

**Authors:** Chongshan Yin, Qicheng Liu, Qing Liu

**Affiliations:** 1 School of Physics and Electronic Science, Changsha University of Science and Technology, Changsha, 410114 China; 2 The Chinese Academy of Sciences, Changsha, 410114 China

**Keywords:** Energy science and technology, Applied physics, Techniques and instrumentation

## Abstract

How to convert heat energy into other forms of usable energy more efficiently is always crucial for our human society. In traditional heat engines, such as the steam engine and the internal combustion engine, high-grade heat energy can be easily converted into mechanical energy, while a large amount of low-grade heat energy is usually wasted owing to its disadvantage in the temperature level. In this work, for the first time, the generation of mechanical energy from both high- and low-temperature steam is implemented by a hydrophilic polymer membrane. When exposed to water vapor with a temperature ranging from 50 to 100 °C, the membrane repeats rolling from one side to another. In nature, this continuously rolling of membrane is powered by the steam, like a miniaturized “steam engine”. The differential concentration of water vapor (steam) on the two sides of the membrane generates the asymmetric swelling, the curve, and the rolling of the membrane. In particular, results suggest that this membrane based “steam engine” can be powered by the steam with a relatively very low temperature of 50 °C, which indicates a new approach to make use of both the high- and low-temperature heat energy.

## Introduction

At the end of the eighteenth century, the introduction of improved steam engine started the classical thermodynamics and the industrial revolution. Until now, how to convert heat energy into other forms of usable energy (such as the mechanical energy and the electric energy) more efficiently has played a crucial role in our human society and economic progress^1–4^. In practice, this conversion of energy mostly relies on the traditional heat engine, such as the steam engine, the internal combustion engine, and the Stirling machine^5–7^. In these heat engines, high-grade heat energy can be easily converted into mechanical energy. However, a large amount of low-grade heat energy is usually wasted, due to its disadvantage in the temperature level^1–8^. For example, in a traditional steam engine, water has to be boiled to produce steam and only the high-temperature and high-pressure steam is available for the generation of mechanical energy^5^. Further, the low-temperature heat energy is rich in variety and huge in quantity all over the world^9–12^, and the utilization of it is of major significance, which is beyond the ability of the traditional heat engines^5–7^. Therefore, new technology to make better use of the heat energy always attracts much attention^1–7,13^. Over more than 200 years, lots of novel heat engines have been invented and developed, for example, the quantum heat engine^14–16^, the osmotic heat engine^17–19^, the miniaturized heat engine^20–26^, the thermoelectric heat engine^27^, the piezo-resistive heat engine^28^, the thermoacoustic Stirling heat engine^29–31^, etc. All of these heat engines show new approach to take advantage of the heat energy worldwide. The advances in the utilization of heat source (especially the low-temperature heat source) will, as always, be important for our modern society.

## Results

### The membrane rolling powered by water vapor

In this work, the generation of mechanical energy from both low- and high-temperature steam is implemented by an asymmetrical shaped polymer membrane. The membrane is prepared based on the per-fluorinated sulfonic acid ionic^32,33^, and details see the “Materials and methods” section. A glass dish of deionized water was placed on a heating plate, and the temperature of water was measured by an infrared sensor and a thermocouple simultaneously. At first, the water was heated to a particular temperature. Then, a water saturated membrane was flatted on the water carefully with a tweezer. Beyond a temperature of 50 °C, the membrane floats on the water and repeats rolling from one side to another. The relative videos are displayed in the Supplementary Information (Video 1 and Video 2 show the membrane rolling on water at 85 °C and 90 °C, respectively). With increasing the temperature from 50 to 90 °C, the membrane rolls faster.

**Figure 1:**
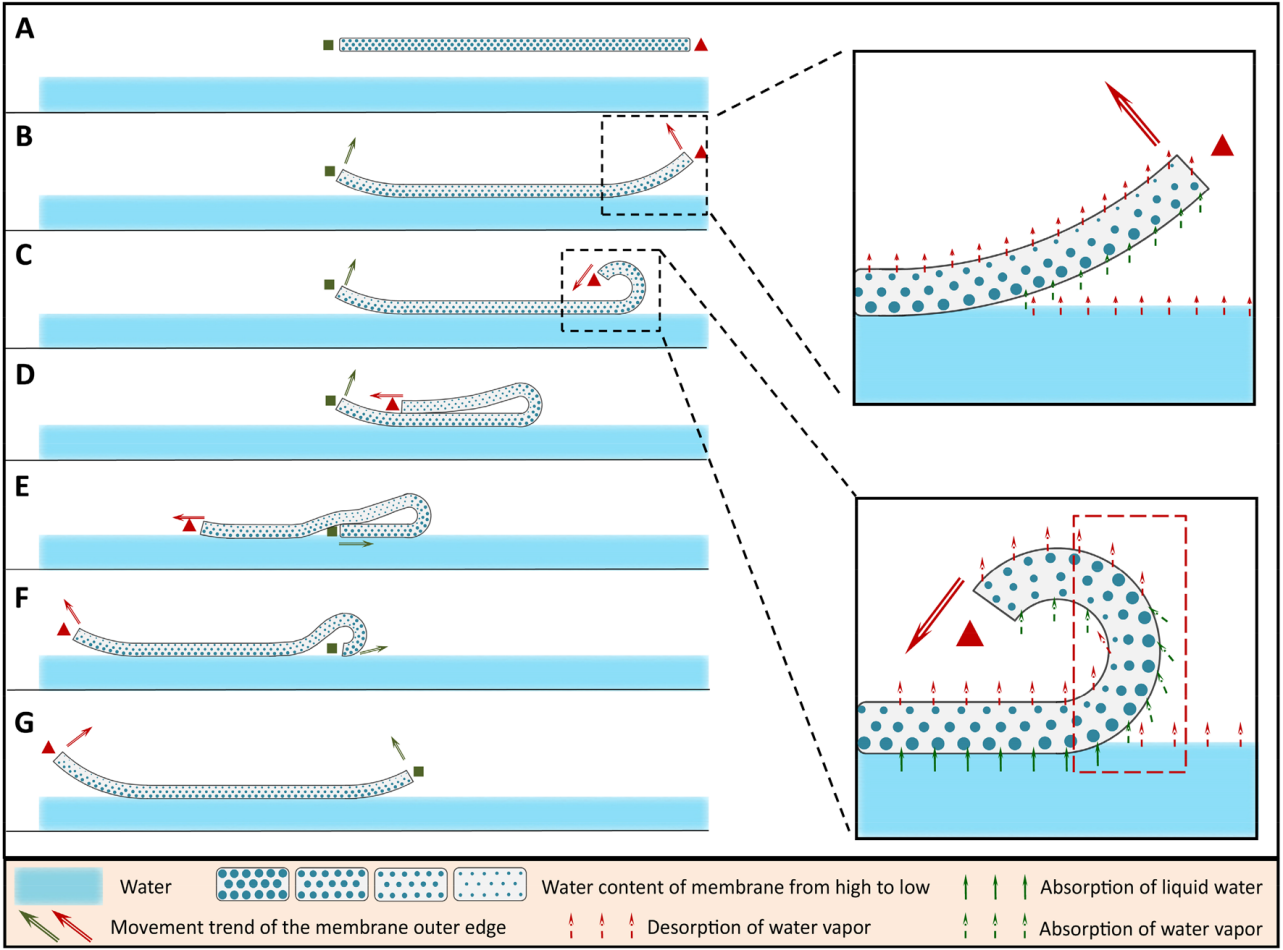
Schematic diagram of the membrane rolling on the water powered by the steam. The rectangle and the triangle are used to mark the left and right outer edges of the membrane, respectively.

The schematic diagram of the membrane rolling is displayed in Fig. 1. As shown in Fig. 1A, the membrane has been water saturated. Figure 1B, the membrane was flatted on the water surface carefully, and the outer edges of the membrane (indicated by the rectangle and the triangle) curve upward. This curving is on account of the shrink (decrease in swelling ratio) of the membrane far water side because of its evaporation of water vapor^32,33^. Figure 1C, one of the membrane outer edges curves inward primarily (the outer edge on the right is employed as an example, marked by the triangle). The bending position of the membrane is denoted with the red dotted box in Fig. 1C. The external surface of the membrane bend shows high swelling ratio because it is exposed to water vapor and is humidified. However, the internal surface of the membrane bend keeps evaporating water and shrinking. Therefore, the asymmetric swelling of the membrane results in the membrane curving and forces the curving to proceed. Figure 1D, the membrane is overlapped owing to a further curving. Figure 1E, the outer edge marked by the triangle continues to move forward until beyond the end of the membrane and contacts the water. Figure 1F, the membrane spreads out on the water. Figure 1G, the left and right of the membrane are reversed, i.e. the membrane completely turns over. These processes then start again and repeat, and the membrane rolling on the water is achieved. In nature, this rolling of membrane is attributed to the difference in the concentration of water vapor (steam) on the two sides of the membrane (the near water side and the far water side), which brings about the differences in water content as well as swelling ratio of the membrane two sides. Physically, this membrane rolling is powered by the water vapor (steam), like a “steam engine”.

**Figure 2:**
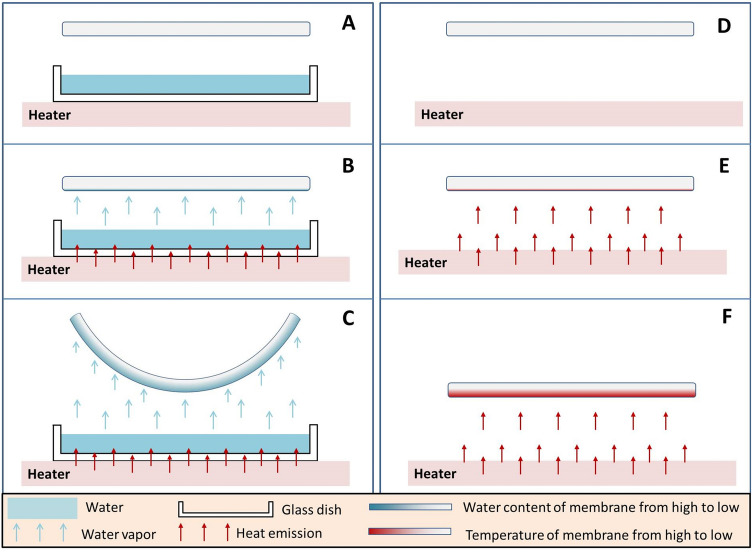
Schematic diagram of the influence of the steam (**A**, **B**, **C**) and the heat (**E**, **F**, **G**) on the dry membrane.

To confirm that the rolling of membrane is powered by the steam, rather than the heat, an additional experiment has been done and shown by the video 3 and Video 4 in the Supplementary Information (Fig. 2 shows the schematic diagram). When put the dry membrane above water vapor (Fig. 2A), numerous water molecules were absorbed by the membrane near water side (Fig. 2B). As a result, the membrane near water side largely swelled and forced the membrane to curve in the direction away from water (Fig. 2C). The Video 3 displays the details. However, when put the dry membrane above the heating plate directly, there is no evident deformation of the membrane (Fig. 2F and Video 4).

**Figure 3:**
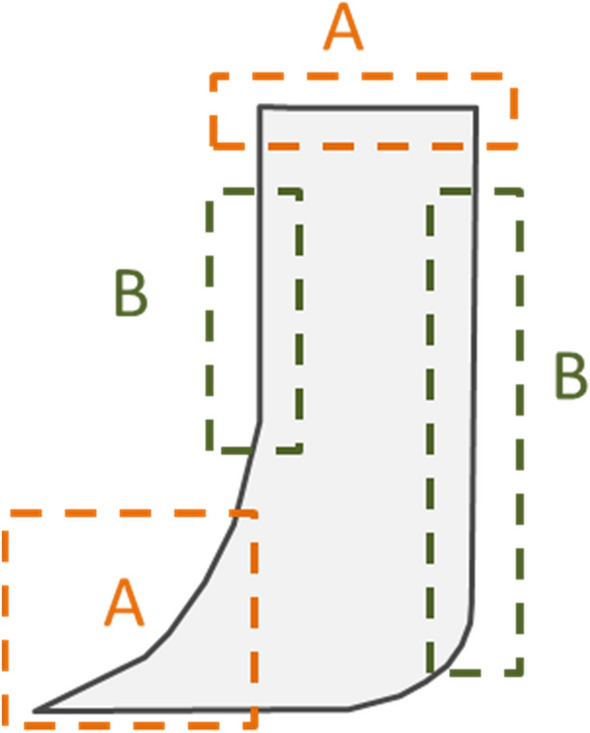
The asymmetrical shape of the membrane. The dotted boxes (**A**) and (**B**) marked the prominent parts and the flat parts of the membrane, respectively.

Figure 3 exhibits the asymmetrical shape of the membrane, and the asymmetrical shape is designed on purpose. Definitely, the curving of the membrane forced by the steam begins from the outer edges. In experiment, for a symmetrical shaped membrane (such as a square or rounded one), its outer edges always curve together and squeeze each other, which highly retards the membrane rolling. However, for an asymmetrical shaped membrane as shown in Fig. 3, the prominent part is more flexible than the flat part, which guarantees that the curving prefers to start from the prominent part, which facilitates the membrane rolling. In addition, even though the prominent parts curve together occasionally, they will stagger with each other and continue the membrane rolling.

### The rolling speed of membrane as a function of water temperature

**Figure 4:**
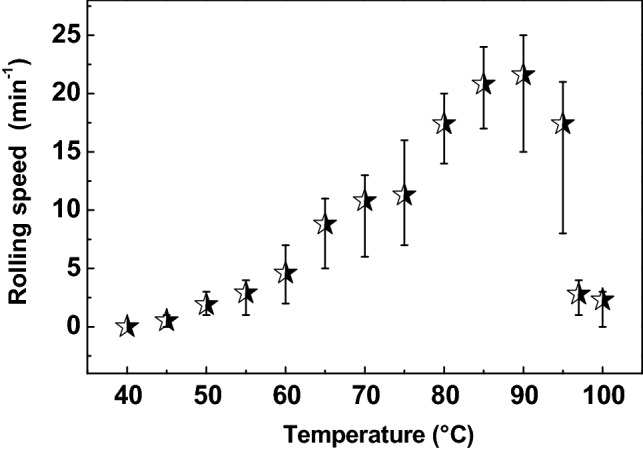
The rolling speed of membrane as a function of water temperature. At each temperature, the measurement has been conducted for 20 times, and the final result is presented as an average value and an error bar.

In the present work, as shown in Fig. 1, the processes from A to G are defined as one time of the membrane rolling. The rolling speed of membrane as a function of water temperature has been measured and displayed in Fig. 4. With increasing the temperature from 50 to 90 °C, the rolling speed of membrane increases. This is caused by the faster evaporation of water from membrane, owing to the higher temperature. As mentioned above, the evaporation of water from the membrane far water side generates the asymmetric swelling, the curving and the continuously rolling of the membrane. Accordingly, the faster evaporation of water facilitates the membrane rolling. In addition, this result confirmed that the membrane rolling is powered by the water vapor.

In particular, the rolling of membrane can be achieved at a relatively very low temperature of 50 °C, which is extremely valuable. To date, most of the world's heat engines are still powered by burning fossil fuels using low-efficiency processes. In a traditional steam engine, water has to be boiled to produce steam, and only the high-temperature steam can be used to generate mechanical energy. However, a large amount of low-grade heat energy in the form of low-temperature and low-pressure steam is wasted^1–4^. Further, the low-temperature heat energy is rich in variety and huge in quantity available worldwide^9–12^. The utilization of this low-temperature heat energy is of major significance^34,35^, which is beyond the ability of traditional steam engine. In the present work, results strongly suggest that the membrane rolling can by powered by the steam with a temperature as low as 50 °C, which indicates a new approach to generate mechanical energy from the abundant low-temperature heat energy in the world.

At a temperature of 90 °C, the average rolling speed of the membrane reaches a maximum value of 21.6 times per minute ( /min). However, further increase in temperature results in a rapid decrement in the rolling speed. Beyond 97 °C, the rolling speed drops to lower than 3 /min, owing to that the bubbling of boiled water disturbed the membrane rolling. The boiled water produced too much steam, which humidified both two sides of the membrane. Thus, the similar swelling ratio of the membrane two sides reduced the asymmetric deformation of the membrane, which is unfavorable to the curving as well as the rolling of the membrane. Accordingly, the present membrane based “steam engine” is capable of the mechanical energy generation from heat source with a temperature ranging from 50 °C to 100 °C, and the generation rate reaches the maximum at 90 °C.

In conclusion, the present work reports a new method to convert heat energy into mechanical energy by means of a polymer membrane. Both the principle and the structure of the membrane based “steam engine” are simple, which indicates low cost of building and operating it. Further, ideally, taking some mechanical energy harvesting technology into consideration, the mechanical energy of the membrane rolling can be converted into other forms of usable energy directly. For example, the triboelectric technology^36–40^ can be a possible approach to make use of the ambient mechanical energy generated by the rolling membrane. As reported by Prof. Wang, Z. L. and co-workers, the film-shaped triboelectric generator is flexible and lightweight, which is based on polymer and can produce electric energy when pressed or warped (mechanical deformation)^39^. Thus, if a triboelectric generator can be attached to the present membrane, the rolling of membrane may drive the triboelectric generator thus realize the conversion of mechanical energy into electrical energy. In addition, similar to the triboelectric generator, the piezoelectric generator^41–43^ is also a possible way for turning the mechanical energy generated by the rolling membrane into electrical energy. These are assumptions, and deeper investigation is needed. Nevertheless, the membrane based “steam engine” may show potential in designing a new-type heat engine.

## Discussion

For the first time, we report a new approach to convert the heat energy into mechanical energy implemented by a hydrophilic polymer membrane. When exposed to the steam with a wide-range temperature from 50 to 100 °C, the membrane repeats rolling from one side to another, like a “steam engine”. In particular, this membrane based “steam engine” can be powered by the steam with a relatively very low temperature of 50 °C, which indicates a new approach to take advantage of the low-temperature heat energy and may show potential in designing a new-type heat engine.

## Methods

The per-fluorinated sulfonic acid ionic was obtained from the purchased commercial Nafion solution (DuPont, DE-520, EW 1100, 5 wt% of perfluorosulfonate resin (H^+^ form) and 95 wt% of isopropanol/water mixture). A certain amount of Nafion solution was transferred into a flat-bottomed quartz dish and evaporated at 60 °C for 8 h. The resulting membranes were thermally treated under vacuum at 135 °C for 2 h^32,33^. The resulted membrane was thoroughly cleaned through being boiled in 5% hydrogen peroxide solution and deionized water for several times. Finally, the membrane was cut into the specially designed asymmetrical shape.

The measurement of membrane rolling speed was conducted under a laboratory environment (25 °C, 50% relative humidity, windless). Before measurement, a glass dish of deionized water was heated to a particular temperature. The temperature was measured by an infrared sensor and a thermocouple simultaneously. Then, a water saturated membrane was flatted on the water carefully with a tweezer. All the membrane rolling speed data was recorded in the first 3 min after introducing the membrane. The measurement was conducted 20 times at each temperature, and the results are presented as the average values.

## Supplementary information

Supplementary file1

Supplementary file2

Supplementary file3

Supplementary file4

Supplementary file5

## Data Availability

The data that supports the findings of this study are available from the corresponding author upon reasonable request.
