# Supplementary material for: Rolling membrane powered by low-temperature steam as a new approach to generate mechanical energy
Source: Sci Rep. 2020 Oct 6;10:16573. doi: 10.1038/s41598-020-73732-7 (PMC7538585; doi:10.1038/s41598-020-73732-7)
Supplement: Supplementary file 1 — Supplementary file1 [file 41598_2020_73732_MOESM1_ESM.docx]

**Supplementary Material**

**Rolling membrane powered by low-temperature steam as a new approach to generate mechanical energy**

Chongshan Yin*^1^, Qicheng Liu^1^, Qing Liu^2^,

1. School of Physics and Electronic Science, Changsha University of Science and Technology, Changsha, 410114, China. *E-mail address: [c.sh.yin@foxmail.com](mailto:c.sh.yin@foxmail.com)

2. The Chinese Academy of Sciences, Changsha, 410114, China.

**Video 1.** The membrane rolling on water at 85 °C.

**Video 2.** The membrane rolling on water at 90 °C.

**Video 3.** When put the dry Nafion membrane above water vapor, the membrane curve in the direction away from water.

#### Video 4. When put the dry membrane above the [heating](javascript:;) [plate](javascript:;) directly, there is no evident deformation of the membrane.

Relative videos are provided along with the Supplementary Material as an attachment (the zip file).

.
